# Usefulness of Stereotactic Radiotherapy Using CyberKnife for Recurrent Lymph Node Metastasis of Differentiated Thyroid Cancer

**DOI:** 10.1155/2017/7956726

**Published:** 2017-03-15

**Authors:** Joji Kawabe, Shigeaki Higashiyama, Mitsuharu Sougawa, Atsushi Yoshida, Kohei Kotani, Susumu Shiomi

**Affiliations:** ^1^Department of Nuclear Medicine, Graduate School of Medicine, Osaka City University, Osaka, Japan; ^2^CyberKnife Center, Osaka Medical College Mishima-Minami Hospital, Osaka, Japan

## Abstract

A woman in her 60s presented with a recurrent lymph node metastasis from a papillary thyroid carcinoma in the right parapharyngeal space. She had already undergone total thyroidectomy, five resections for cervical lymph node metastases, and right carotid rebuilding. Surgical resection of the current metastasis was impossible. ^131^I-radioiodine therapy (RIT) with 3.7 GBq ^131^I was not effective; therefore, stereotactic radiation therapy (SRT) using a CyberKnife radiotherapy system was scheduled. The prescription dose was 21 Gy, and a dose covering 95% of the planning target volume (PTV) in three fractions was administered. The PTV was 4,790 mm^3^. Follow-up magnetic resonance imaging conducted 3 and 12 months after the SRT demonstrated a remarkable and gradual reduction of the recurrent lymph node metastasis in the right parapharyngeal space and no evidence of recurrence. For multidisciplinary therapy of unresectable and/or RIT unresponsive locoregional lymph node metastases and recurrences of DTC, SRT using the CyberKnife system should be considered.

## 1. Introduction

The application of external beam radiotherapy (EBRT) as postoperative treatment for differentiated thyroid cancer (DTC) has been controversial in the field of head and neck surgery, as well as in radiation oncology [1–3]. We present a case of recurrent lymph node metastasis of DTC treated effectively with stereotactic radiation therapy (SRT) by using a CyberKnife radiotherapy system (Accuray Inc., Sunnyvale, CA, USA) [4, 5]. Written informed consent was obtained from the patient for publication of this case report and accompanying images are in accordance with the Code of Ethics of the World Medical Association.

## 2. Case Report

**Figure 1:**
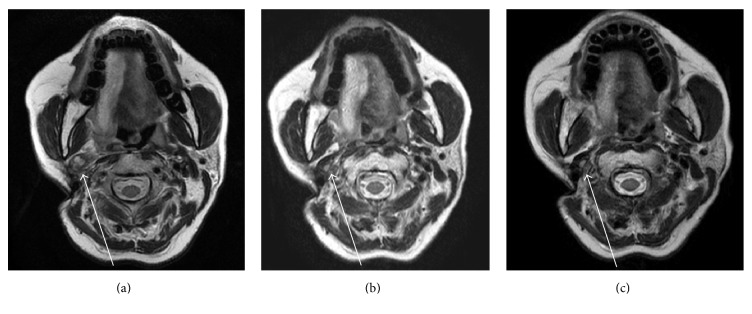
Magnetic resonance T2-weighted imaging findings. (a) Postsurgical status: a recurrent lymph node metastasis from a papillary thyroid carcinoma in the right parapharyngeal space is shown (white arrow). (b) Three months after stereotactic radiation therapy (SRT) using the CyberKnife radiotherapy system, the lymph node metastasis is remarkably reduced. (c) Twelve months after SRT, the reduced lymph node metastasis has not changed. No recurrences were recognized, which indicates therapeutic effectiveness.

**Figure 2:**
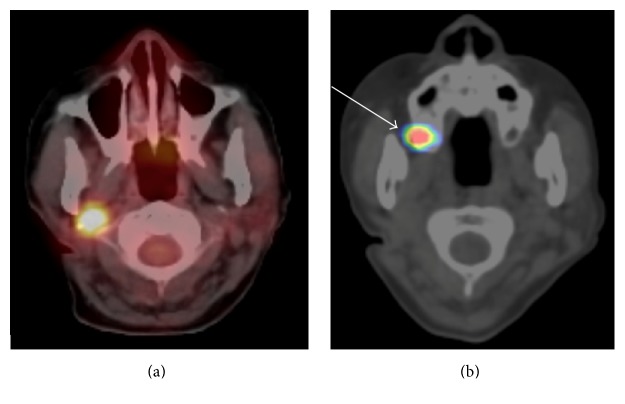
(a) ^18^F-FDG PET/CT image: remarkable abnormal FDG uptake by the lymph node metastasis in the right parapharyngeal space. (b) ^131^I SPECT/CT image obtained 7 days after a ^131^I 3.7 GBq dose of RIT: ^131^I uptake by the lymph node metastasis is not evident. The abnormal distribution in the right maxillary sinus (white arrow) represents ^131^I secretion into the nasal fluid.

A woman in her 60s presented with a recurrent lymph node metastasis in the right parapharyngeal space from a papillary thyroid carcinoma (Figure 1(a)). After undergoing right lobectomy of the thyroid and right neck dissection 7 years ago, she experienced five episodes of recurrences of cervical lymph node metastases. Resections of the metastases, left thyroid lobectomy, and right carotid rebuilding were performed. At this time, highly abnormal ^18^F-fluorodeoxyglucose (FDG) uptake was detected in the right parapharyngeal space, which was diagnosed as a recurrent lymph node metastasis (Figure 2(a)). Surgical resection was thought to be difficult due to postsurgical severe changes and carotid rebuilding.

She was referred to our hospital for radioiodine therapy (RIT), which comprised of 3.7 GBq of ^131^I. However, the lymph node metastasis did not take up the ^131^I (Figure 2(b)). Therefore, the RIT was thought to be ineffective, and EBRT was considered. SRT using the CyberKnife radiotherapy system involves the precise delivery of high dose radiation stereotactically to a target in a small number of fractions. The steep dose fall-off minimizes the radiation to surrounding tissues beyond a few millimeters [4].

**Figure 3:**
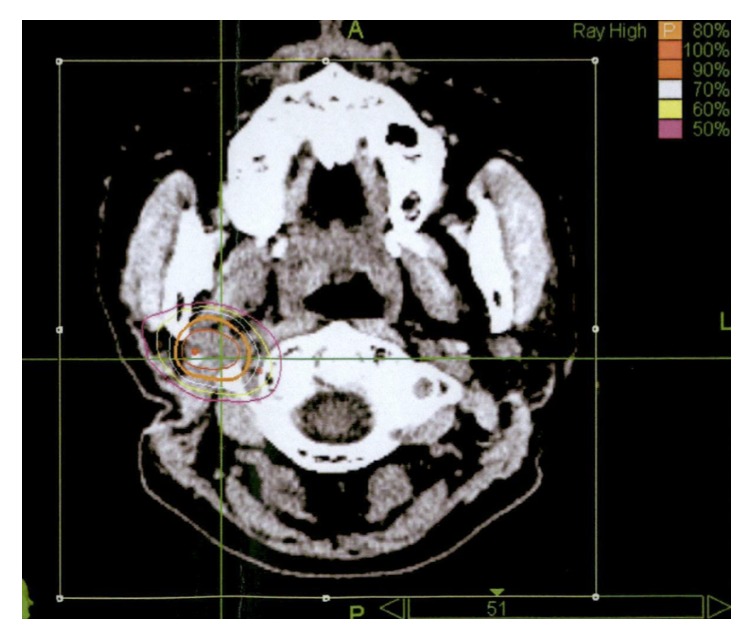
Dose distribution for the lymph node metastasis in the right parapharyngeal region. The planning target volume is illustrated by the thin orange line.

A prescription dose of 21 Gy and a dose covering 95% of the planning target volume (PTV) (and the maximum dose of 26.3 Gy) were administered in three fractions at Osaka Medical College Mishima-Minami Hospital CyberKnife Center. The PTV was 4,790 mm^3^ (Figure 3). The total dose and fractional dose were determined according to the tumor volume and the patient's general condition. Acute adverse events were not observed.

Follow-up magnetic resonance imaging performed 3 and 12 months (Figures 1(b) and 1(c)) after the SRT demonstrated a remarkable and gradual reduction in the recurrent lymph node metastasis in the right parapharyngeal space and no evidence of recurrence.

## 3. Discussion

Generally, postsurgical treatment of DTC patients with metastases involves RIT [2, 3], and the use of EBRT has been controversial [1–3]. Ford et al. reported that EBRT requires a total dose of at least 50 Gy and possibly higher, in addition to RIT, in order to have any impact on local control [1]. Acute (mucositis, dysphagia, skin reactions, and edema) and late (skin fibrosis and tracheal compression) reactions to EBRT have been reported [6–8]. SRT delivers high radiation doses to small lesions with short fractionation schemes under the most stringent conditions, allowing for high dose conformity and sparing of healthy tissue. This should help to overcome the long-term toxicity concerns about conventional EBRT [9].

In our case, surgical resection of the lymph node metastasis in the right parapharyngeal space was impossible. Therapeutic effectiveness of RIT could not be expected. Therefore, we administered SRT using the CyberKnife system, which allowed for a reduction in the lymph node metastasis. A year after the SRT, no recurrence has been detected.

SRT using the CyberKnife system demonstrated good therapeutic performance in our case. The prescribed dose in our case was 21 Gy. Yamazaki et al. reported the prescribed dose to head and neck tumor lesions, including cervical lymph node metastases, ranged from 13 Gy to 39 Gy, with a median of 30 Gy [10]. Therefore, a total dose of 21 Gy was thought to be appropriate.

In 2015, the American Thyroid Association Management Guidelines for Adult Patients with Thyroid Nodules and Differentiated Thyroid Cancer (the ATA guideline) considers using EBRT delivered via modern techniques such as stereotactic radiation for locoregional recurrence that is not surgically resectable. However, as far as we know, no previous report of treating DTC with SRT by using a CyberKnife system has been published.

## 4. Conclusion

For multidisciplinary therapy of unresectable and/or RIT ineffective locoregional lymph node metastases and DTC recurrence, SRT delivered via the CyberKnife system should be considered.
